# High-Throughput 13-Parameter Immunophenotyping Identifies Shifts in the Circulating T-Cell Compartment Following Reperfusion in Patients with Acute Myocardial Infarction

**DOI:** 10.1371/journal.pone.0047155

**Published:** 2012-10-16

**Authors:** Jedrzej Hoffmann, Karel Fiser, Jolanta Weaver, Ian Dimmick, Monika Loeher, Hanspeter Pircher, Carmen Martin-Ruiz, Murugapathy Veerasamy, Bernard Keavney, Thomas von Zglinicki, Ioakim Spyridopoulos

**Affiliations:** 1 Institute of Genetic Medicine, Newcastle University, Newcastle, United Kingdom; 2 Institute of Ageing and Health, Newcastle University, Newcastle, United Kingdom; 3 Institute of Cellular Medicine, Newcastle University, Newcastle, United Kingdom; 4 Department of Immunology, Institute of Medical Microbiology and Hygiene, Freiburg University, Freiburg, Germany; 5 CLIP – Childhood Leukaemia Investigation Prague, Department of Paediatric Haematology and Oncology, 2nd Medical School, Charles University Prague, Prague, Czech Republic; Albert Einstein College of Medicine, United States of America

## Abstract

**Rationale:**

With the advent of primary PCI (PPCI), reperfusion is achieved in almost all patients presenting with acute myocardial infarction. However, despite multiple trials, reperfusion injury has not been successfully dealt with so far. In mouse models, CD4**^+^** T lymphocytes (T cells) have been shown to be crucial instigators of reperfusion injury.

**Objective:**

Our goal was to investigate the role of CD4**^+^** T cells during myocardial reperfusion following PPCI by developing a protocol for high-throughput multiplexed flow cytometric analysis and multivariate flow clustering.

**Methods and Results:**

13-parameter immunophenotyping and hierarchical cluster analysis (HCA) identified a unique CD4**^+^**CD57**^+^** T-cell population in PPCI patients that reflected acute proliferation in the CD4**^+^** T-cell compartment. CD4**^+^**CCR7**^+^** T cells were specifically depleted from peripheral blood during the first 30 min of myocardial reperfusion after PPCI, suggesting a potential role for the chemokine receptor CCR7 in T-cell redistribution to either peripheral tissues or migration to the infarcted heart during ischemia/reperfusion following PPCI.

**Conclusions:**

High-throughput polychromatic flow cytometry and HCA are capable of objective, time and cost efficient assessment of the individual T-cell immune profile in different stages of coronary heart disease and have broad applications in clinical trials.

## Introduction

In patients with acute ST-elevation myocardial infarction (STEMI), early and successful reopening of the infarct related coronary vessel by primary percutaneous coronary intervention (PPCI) is the most effective treatment strategy proven so far. Myocardial reperfusion, however, can induce myocardial injury and cell death. Neutrophil granulocytes, migrating into the myocardium, are believed to mediate cardiomyocyte death by microvascular obstruction (MVO) and generation of ROS (reactive oxygen species). MVO is associated with larger infarct size, adverse left ventricular remodelling and more frequent adverse cardiovascular outcomes [1]. In patients with STEMI treated with PPCI, up to 40% of patients have impaired microvascular perfusion [2]–[4]. Nonetheless, the treatment of MVO, which is associated with the “no-reflow” phenomenon, remains elusive. Studies in animal models of acute myocardial infarction suggest that reperfusion injury is a major determinant of final infarct size. In patients with STEMI, PPCI has reduced in-hospital mortality to as low as 5%. However, a significant proportion of patients will suffer from post-infarction heart failure or die in due course from complications related to left ventricular dysfunction. Therefore, future therapies for STEMI have to target causes of post-infarction heart failure, including myocardial reperfusion injury.

Mouse studies have generated evidence that in kidney, lung and myocardial ischemia-reperfusion (I/R) injury T lymphocytes (T cells) are the important effectors [5]–[7]. Studies in a mouse model of I/R have revealed that CD4**^+^** T cells, but not CD8**^+^** T cells, invade the infarcted myocardium, triggering the influx of neutrophils from the bone marrow [8], [9]. The role of T cells in acute reperfusion of STEMI in humans has not been investigated in detail so far. Two studies have shown the adverse prognostic relevance of lymphopenia during the first 96 h of STEMI [10], [11]. However, no study so far has looked into the regulation of specific lymphocyte populations during STEMI and especially the reperfusion phase. This is becoming increasingly more important due to the widespread use of PPCI especially in North America and the UK, where thrombolytic therapy has been replaced as the treatment of choice over the last 4 years.

In this study, we describe a novel detailed protocol for immunostaining and high-throughput multiparameter flow cytometric analysis of peripheral blood T-cell subsets and its potential applications for a multicenter clinical trial. By using an extended panel of monoclonal antibodies targeting differentiation and senescence surface markers, we were able to dissect out immunophenotypic profiles of several T-cell subsets in patients with acute myocardial infarction following PPCI. Application of a novel multivariate-clustering algorithm enabled an *a priori* objective screening to identify differences in immunophenotypic and functional T-cell profiles in patients with coronary heart disease.

## Materials and Methods

Please see online Materials S1 for further details.

### Ethics Statement

The study protocol was approved by the institutional ethical committee of Newcastle University (REC 09/H0905/50), and written informed consent was obtained from all patients and healthy volunteers.

### Study subjects

55 men with angiographically confirmed coronary heart disease were included into the study. 31 patients with acute ST-elevation myocardial infarction (STEMI, mean age 56.2±5.6 years) and 24 stable patients with healed STEMI (mean time 6 months after infarction, mean age 61±6 y) were analysed 24 hours following primary coronary intervention (PPCI) or routine coronary angiography, respectively (Table 1). None of the patients were affected by neoplastic, autoimmune or chronic infectious disease. All subjects with recent infections were also excluded. 18 healthy male volunteers were enrolled as controls (mean age 53.5±5.8 y). For the time-course additional 17 patients with STEMI (mean age 61.5 years) were sequentially analysed within 48 hours following PPCI (Table 2).

**Table 1:** Clinical patient characteristics of the main study group.

	STEMI, 24 h post PCI	Stable, >3 months post STEMI
n	31	24
Age, years, median (range)	58.1 (45–65)	61.8 (47–74)
Sex: M/F	31/0	24/0
Initial troponin I, ng/L	7.4±2.9	NA
Peak troponin I, ng/L	35.7±3.3	NA
CK, U/L	420±653	NA
Number of vessels (1/2/3)	19/7/5	6/4/14
TIMI flow before PCI (0/1/2/3)	26/0/4/1	NA
Onset-to-balloon time, min	194±28	NA
Door-to-balloon time, min	26±2	NA
Ejection fraction, %	46±2*	38±4
**Risk factors**		
HDL cholesterol, mmol/L	1.1±0.04	4.2±0.05
LDL cholesterol, mmol/L	3.9±0.2	3.1±0.3
Triglycerides, mmol/L	1.6±0.2	2.1±0.2
Dyslipidemia	7/31	11/24
BMI	28.7±0.9	29.4±1.3
Diabetes	3/31	8/24
Hypertension	7/31	14/24
Current smoking	14/31	3/31
Family history of CHD	18/31	11/24
**History**		
Previous MI	1/31	24/24
Previous CABG	0/31	6/24
Previous PCI	2/31	24/24
Statins	6/31	23/24

CABG: coronary artery bypass graft; PCI: percutaneous coronary intervention; CHD: coronary heart disease; MI: myocardial infarction; NA: not applicable.

* For STEMI patients ejection fraction 3 days after event.

**Table 2:** Clinical patient characteristics of the time course substudy group.

	STEMI, time course substudy
n	17
Age, years, median (range)	63 (48–78)
Sex: M/F	10/7
Troponin T on admission, ng/L	100±29
Troponin T at 12 h, ng/L	3201±839
CK, U/L	133±19
Number of vessels (1/2/3)	17/0/0
TIMI flow before PCI (0/1/2/3)	13/0/2/2
Onset-to-balloon time (min)	196±20
Door-to-balloon time (min)	28±3
Ejection fraction, %	47±3
**Risk factors**	
HDL cholesterol (mmol/L)	1.4±0.1
LDL cholesterol (mmol/L)	4.0±0.3
Triglycerides (mmol/L)	1.8±0.3
Dyslipidemia	2/17
BMI	26.7±1.4
Diabetes	0/17
Hypertension	5/17
Current smoking	10/17
Family history of CHD	11/17
**History**	
Previous MI	2/17
Previous CABG	0/17
Previous PCI	2/17
Statins	1/17

CABG: coronary artery bypass graft; PCI: percutaneous coronary intervention; CHD: coronary heart disease; MI: myocardial infarction; NA: not applicable.

### Blood Collection and enumeration of peripheral blood leukocyte subsets

80 ml or 20 ml (for the time-course substudy) of peripheral blood were drawn into EDTA collection tubes. Absolute counts of peripheral blood granulocytes, monocytes and lymphocyte subpopulations were determined directly using 5-colour BD TruCount flow cytometry assay (BD Biosciences).

### PBMCs isolation, cell cryopreservation and storage

Peripheral blood mononuclear cells (PBMCs) were obtained after Ficoll density gradient centrifugation using Ficoll-Hypaque. Following isolation, PBMCs were counted using a Neubauer haemocytometer, carefully resuspended in ice-chilled freezing medium containing RPMI 1640 with 10% FBS, 1% P/S and 10% DMSO (100 µl medium pro 1×10^6^ cells) and aliquoted into 1 ml cryovials. Cell aliquots were frozen at −80°C using 1°C freezing containers with isopropyl alcohol (Thermo Fisher Scientific), and stored at −80°C for no longer than 6 months.

### Cell staining for 11-colour flow cytometry and flow cytometry data acquisition

Frozen PBMC aliquots were quickly thawed, and washed once using automatic cell washer. Following primary wash step, cell viability and cell numbers were analysed using Vi cell counter. 1×10^6^ PBMCs were then incubated with the primary antibody mix for 20 minutes at RT in the dark. Following three wash steps, the secondary conjugates and Aqua dead cell dye (Invitrogen) were added. Samples were incubated in the dark for another 20 minutes at RT. Following final wash steps unfixed samples were immediately subjected to analysis by flow cytometry. Samples were measured in our flow core facility on a BD LSR II cytometer (see supplementary material for detailed description) using BD FACSDiva acquisition software. PMT voltages and compensation values were set as described below. At least 100.000 viable cell events per sample were acquired.

### Flow cytometry data analysis

The analysis of acquired data was performed using MATLAB (for Hierarchical Cluster Analysis, see below) or FlowJo software (Version 9.4.1 for Macintosh). Two-dimensional plots (pseudo-color or dot plot) were created using biexponential transformation and sequential gating of viable T cells was performed according to the model of T-cell memory differentiation as described by Sallusto and Romero [12], [13].

### Hierarchical Cluster Analysis (HCA) of flow cytometry data

Following data acquisition, files were exported from DIVA and saved as .fcs version 3.0 files. The analysed parameters were eleven colour channels (KLRG1, CD3, CD28, CCR7, CD45RA, CD57, CD27, CD4, CD8, PD1, Aqua Dye) and 3–4 parameters based on forward and side scatters (FS-A, SS-A, SS-H, (SS-W)). The raw or CD3**^+^** positive cells pre-gated data were extracted from .fcs files and imported into MATLAB environment (MATLAB, MathWorks, Natick, MA), where all subsequent steps were carried out. Compensation matrix (as present in .fcs files) was applied to the data followed by biexponential-like transformation and normalisation (zscore). After this the data were ready for HCA, which was performed using our new algorithm described previously [14].

### 6-colour flow sorting of CD4 T Cells

3×10^7^ cryopreserved PBMCs were used for fluorescence-activated cell sorting. PBMC staining was performed using anti-human-CD3-FITC, CD4-PE-Cy7, CD8-APC-H7, CCR7-PE, CD45RA-Pacific Blue and Aqua dead cell dye. Cell sorting was performed in our flow core facility on a BD FACS Aria II cell sorter. The sorted aliquots were spun down and the flow buffer supernatant was completely removed with a vacuum suction. Dry T-cell pellets were stored at −80°C freezer until further processed with DNA isolation protocol (see below).

### DNA isolation and telomere length RT-PCR assay

DNA was extracted from sorted CD4**^+^** T cells with the QIAamp DNA Mini Kit (Cat. No. 51304, Qiagen Ltd, Crawley, UK). Telomere length was measured by quantitative real-time polymerase chain reaction (PCR) with modifications as described previously [15].

### Statistical analysis

In the text, data are reported as mean±SE. Comparison of 3 means was performed by ANOVA, followed by Tukeys' post-hoc test. Comparison of 2 groups was calculated using an unpaired t-test, if normal probability plots (P-P plots) demonstrated approximate normality. All statistical tests were performed using GraphPad Prism version 5 for Macintosh (www.graphpad.com).

## Results

### CD4 central memory T cells are elevated during the first 24 h after PPCI

**Figure 1:**
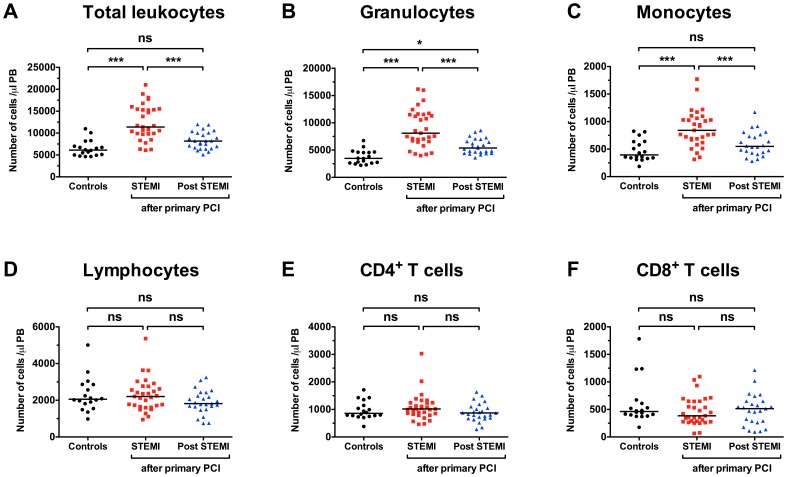
Peripheral leukocytosis after primary percutaneus coronary intervention (PPCI). Absolute number of total leukocytes, neutrophil granulocytes and monocytes (**A**), as well as lymphocytes and the main T-cell subsets (**B**) in peripheral blood. Concentration of leukocyte populations are displayed for patients 24 h following PPCI in STEMI (n = 31), 3 months post PPCI (n = 24), and in age-matched healthy Controls (n = 18). 1-way ANOVA with Tukeys' post-hoc test was performed. *p<0.05, ***p<0.001, ns not significant.

**Figure 2:**
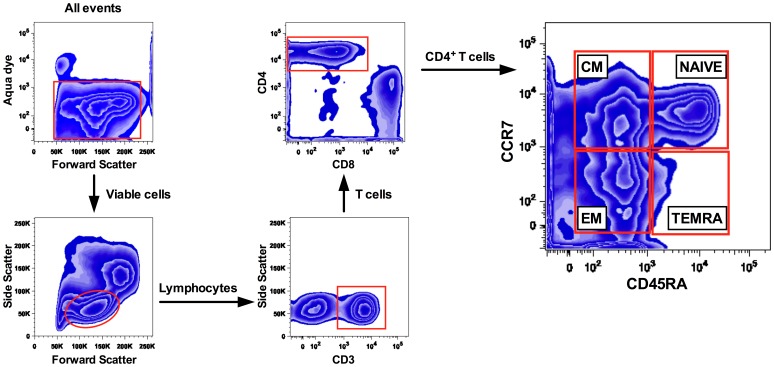
Basic gating strategy for CD4^+^ T cells. Viable cells, as distiguished on the basis of Aqua dye staining were gated and CD4**^+^** T cells were selected based on their light scatter properties and CD3 and CD4 co-expression. CD4**^+^** T-cell subpopulations were separated into main subsets based on the CD45RA/CCR7 expression properties, as previously described by Sallusto et al.: NAIVE (CD45RA**^+^**CCR7**^+^**), CENTRAL MEMORY (CD45RA**^−^**CCR7**^+^**), EFFECTOR MEMORY (CD45RA**^−^**CCR7**^−^**) and CD45RA reexpressing „revertant” memory CD4**^+^** T cells (T effector memory CD45RA**^+^** cells, TEMRA). Further sequential gating analysis were perfomed based on the coexpression of co-stimulatory markers CD27/CD28 as well as proliferation/senescence markers CD57, KLRG1, and PD1.

**Figure 3:**
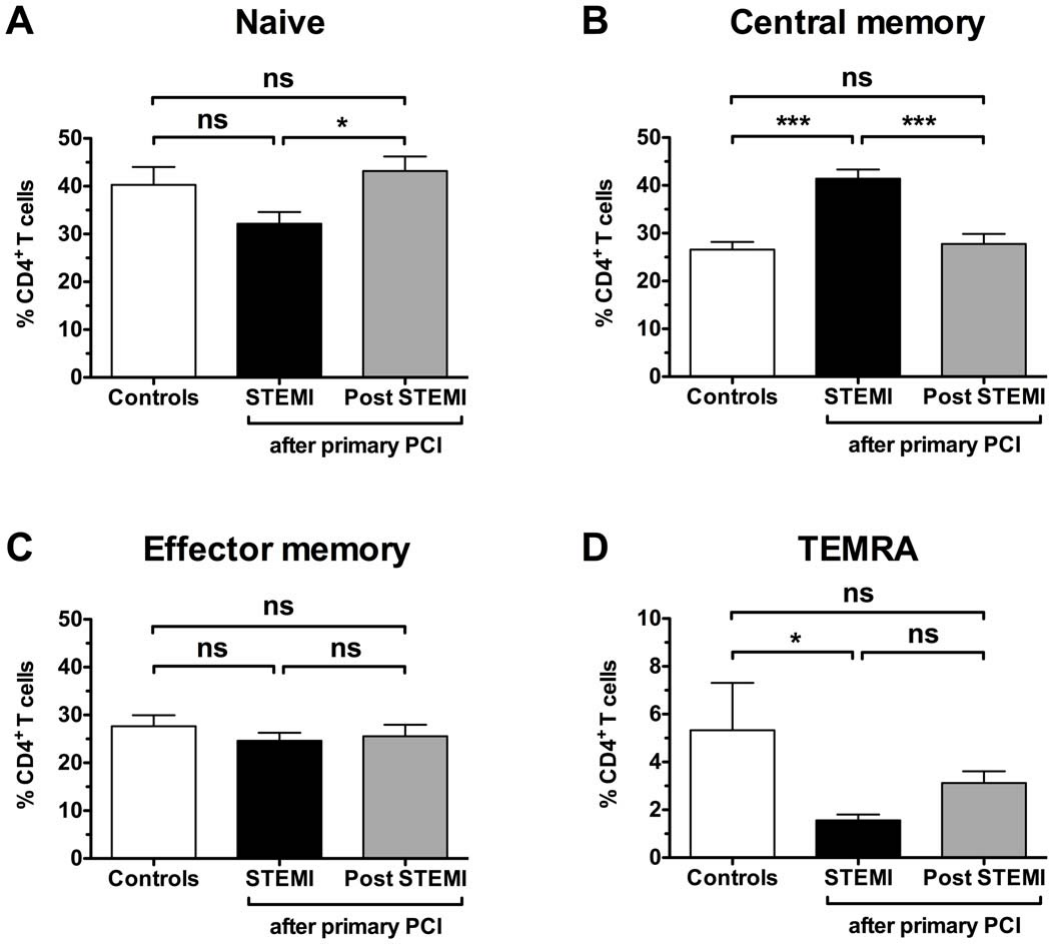
Distribution of main CD4 T-cell subsets. Frequencies of CD4**^+^** naive (CD45RA**^+^**CCR7**^+^**), central memory (CD45RA**^−^**CCR7**^+^**), effector memory (CD45RA**^−^**CCR7**^−^**) and terminally differentiated effector memory TEMRA cells (CD45RA**^+^**CCR7**^−^**) are shown. Groups are identical to Figure 1. 1-way ANOVA with Tukeys' post-hoc test was performed. *p<0.05, ***p<0.001, ns not significant.

To investigate the pattern of CD4**^+^** T cells following PPCI, we compared leukocyte subsets from 18 age-matched healthy controls to patients with acute myocardial infarction following PPCI (24 h, n = 31 and 6 months, n = 24) (Figure 1). While total leukocytes (12418±3969 vs. 6502±1845 cells/µl, p<0.001 vs. control), granulocytes (8987±3448 vs. 3732±1277 cells/µl, p<0.001 vs. control) and monocytes (873±330 vs. 473±191 cells/µl, p<0.001 vs. control) were all elevated 24 h following PPCI (Figure 1A), no significant changes appeared in the lymphocyte population or CD4 subpopulation, respectively (Figure 1B). At 6 months following PPCI, only granulocytes appeared slightly elevated (5700±1300 vs. 3700±1300 cells/µl, p<0.05 vs. control). Further separation of CD4**^+^** T cells into naïve, central memory (CM), effector memory (EM) and terminally differentiated EM cells (TEMRA) was performed by adding antibodies for the chemokine receptor CCR7 and CD45RA (Figure 2). Surprisingly, a 56% relative increase in central memory cells compared to the control group was now apparent at the 24 h time point (41±10% vs. 27±7%, p<0.001, Figure 3). 6 months post PPCI this increase was no longer detectable (28±10%, p<0.001 vs. 24 h, Figure 3). Parallel to the relative increase of CD4**^+^** CM cells we saw a decrease in naive and TEMRA cells (Figure 3). Thus, these results indicate a specific shift within the CD4**^+^** T-cell population as the result of either infarct vessel occlusion or reperfusion by PPCI.

### 13-parameter immunophenotyping and hierarchical cluster analysis identifies a unique CD4^+^CD57^+^ T-cell population in STEMI patients

We have developed a protocol, which allows for accurate 13-parameter polychromatic analysis of cryopreserved mononuclear cells even months after cell isolation. To test inter-assay variability, we processed cryopreserved samples from the same donor on 27 different days (Table 4 in Materials S1). CV for % CD4**^+^** out of all T cells was 5.8%, and CVs for the 3 main populations (naïve, CM and EM) of CD4 T cells between 6 and 9%. Of note, CVs of absolute CD4**^+^** and CD8**^+^** T-cell numbers from fresh blood were below 2% (data not shown).

**Figure 4:**
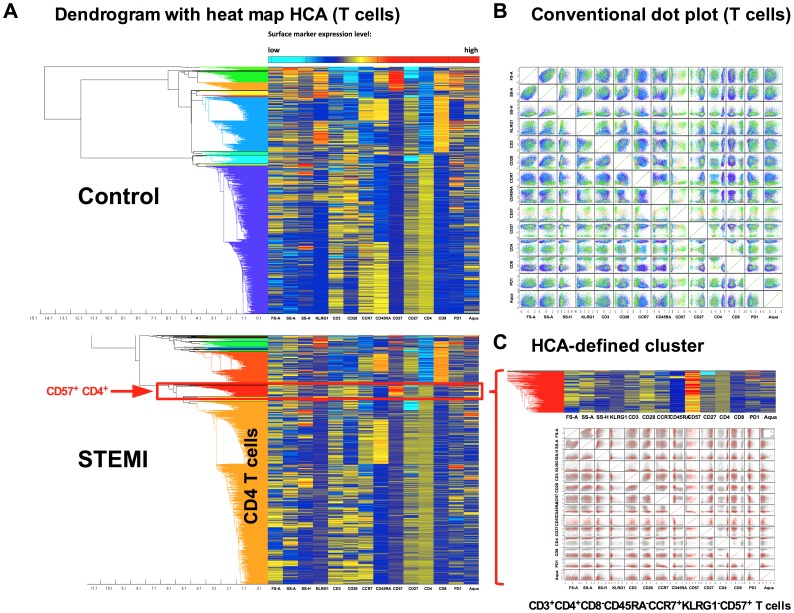
Hierarchical clustering analysis of 13-parameter flow cytometry data. Dendrogram with heatmap-HCA of 10^4^ events (gated on viable CD3^+^ T cells) acquired from PBMCs of healthy control and STEMI patient (24 h after PPCI). Heatmap shows relative levels of all 13 parameters (columns) in all 10^4^ events (rows) in color coding (blue, low expression; red, high expression). Dendrogram shows the hierarchy of T cells based on their similarity in all parameters measured. Colored branches of the dendrogram are selected clusters (A). The T-cell subsets from healthy controls (as identified by HCA) are displayed on conventional scatter dot plots (B). HCA revealed that the proliferation marker CD57 is strongly expressed on CD4**^+^** CM T cells in STEMI patients (zoom on cluster of interest in C).

After processing all 73 cryopreserved samples from controls and patients, hierarchical clustering analysis (HCA) was used to visualize differential cell populations between patients and controls. HCA builds a hierarchical tree of nested clusters (populations) based on their similarities (Figure 4A). This hierarchical tree (or dendrogram) is drawn next to a heatmap table. The identified clusters can be additionally plotted on conventional two-dimensional dot blots (Figure 4B). In our study population, we could identify a CD4**^+^**CD57**^+^** cluster among all T cells that appeared specific for patients following PPCI (Figure 4A). This HCA-defined CD4**^+^**CD57**^+^** cluster is characterized by CD3**^+^**CD4**^+^**CD8**^−^**CD45RA**^−^**CCR7**^+^**KLRG1**^−^**CD57**^+^** staining (all two-parameter combination plots of a defined cluster are shown in Figure 4 C). In terms of T-cell subpopulations, this identifies CD4**^+^** central memory cells that are staining positive for CD57 and negative for KLRG1.

### Increased expression of CD57 in central memory cells indicates acute proliferation in the CD4 T-cell compartment

**Figure 5:**
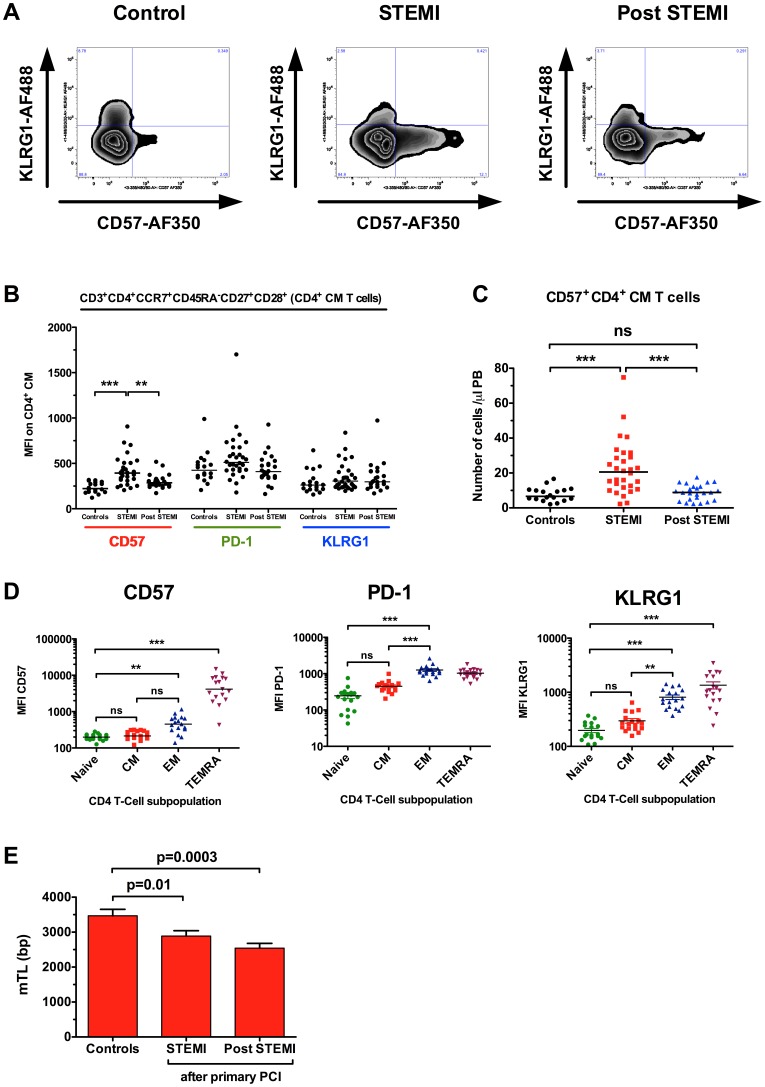
Increased expression of CD57 in central memory cells indicates acute proliferation in the CD4 T-cell compartment. Conventional gating analysis confirmed increased expression of proliferation associated markers CD57 on CM CD4**^+^** T cells of STEMI pts (MFI – mean fluorescence intensity). This was reflected by significantly increased frequencies of specific KLRG1**^−^**CD57**^+^** CD4 CM**^+^** T cells (A–C). The validity of CD57, PD-1 and KLRG1 as proliferation-associated markers was determined in healthy controls according to Sallusto's model of T-cell differentiation (D). Mean telomere length (mTL) in CD4**^+^** T cells is reduced in patients with STEMI when compared to age-matched controls (E). *p<0.05, **p<0.01, ***p<0.001, ns not significant.

Both CD57 and KLRG1 have been dubbed senescence markers, however, CD57 expression on CD4**^+^** T cells is a general marker of proliferative inability, a history of more cell divisions, and short telomeres [16]. The lack of KLRG1 expression in the CD57**^+^** CM subpopulation indicates very recent cell proliferation without reaching senescence (Figure 5A) [16]–[18]. Next we compared the mean fluorescence intensity (MFI) for 3 different proliferation/senescence markers in CD4**^+^** CM cells. While MFI for CD57 increased from 235±60 to 419±160 in patients 24 h post PPCI (Figure 5B, p<0.001), MFI for PD-1 and KLRG1 remained unchanged. This strongly suggests a specific upregulation of CD57 secondary to proliferation. To further lend proof to this hypothesis, we measured MFIs in the 4 major subpopulations of CD4**^+^** T cells from healthy controls (Figure 5C). CD57 MFI for naive CD4**^+^** cells (202±39) and CM cells (232±61) did not differ, while MFI in EM (506±263, p = 0.01 vs. naive) and TEMRAs (5571±3928, p = 0.001 vs. naive) was significantly higher than in naive cells. This analysis strongly suggests, that the increase in CD57 MFI in PPCI patients is not simply due to a shift in subpopulations, but the result of a real increase in numbers of completed cell divisions. Interestingly, while CD57 MFI seems to distinguish very well between EM and TEMRAs (p = 0.001), PD-1 and KLRG1 separate CM and EM populations well (Figure 5C). To document that the increase in CD4**^+^**CD57**^+^** CM T cells is not just a relative shift, we analysed the total number of cells in peripheral blood. Here we found an increase of CD4**^+^**CD57**^+^**KLRG1**^−^** central memory cells from 8±4 cells/µl to 23 cells/µl (p<0.001, Figure 5D). Finally, CD4**^+^** T cells were sorted from cryopreserved mononuclear cells by flow cytometry and mean telomere length (mTL) was determined by real time PCR from all study participants. Patients with acute myocardial infarction had significant shorter mTL in their CD4**^+^** T cells 24 h after PPCI (2.9±0.9 vs. 3.5±0.8 kB, p = 0.01) when compared to healthy age-matched controls (Figure 5E). 6 months post PPCI, mTL of CD4**^+^** T cells appeared even shorter (2.5 kB, p = 0.003 vs. control), suggesting further cell divisions in this cell compartment.

### CD4^+^CCR7^+^ T cells are specifically depleted from peripheral blood during myocardial reperfusion

**Figure 6:**
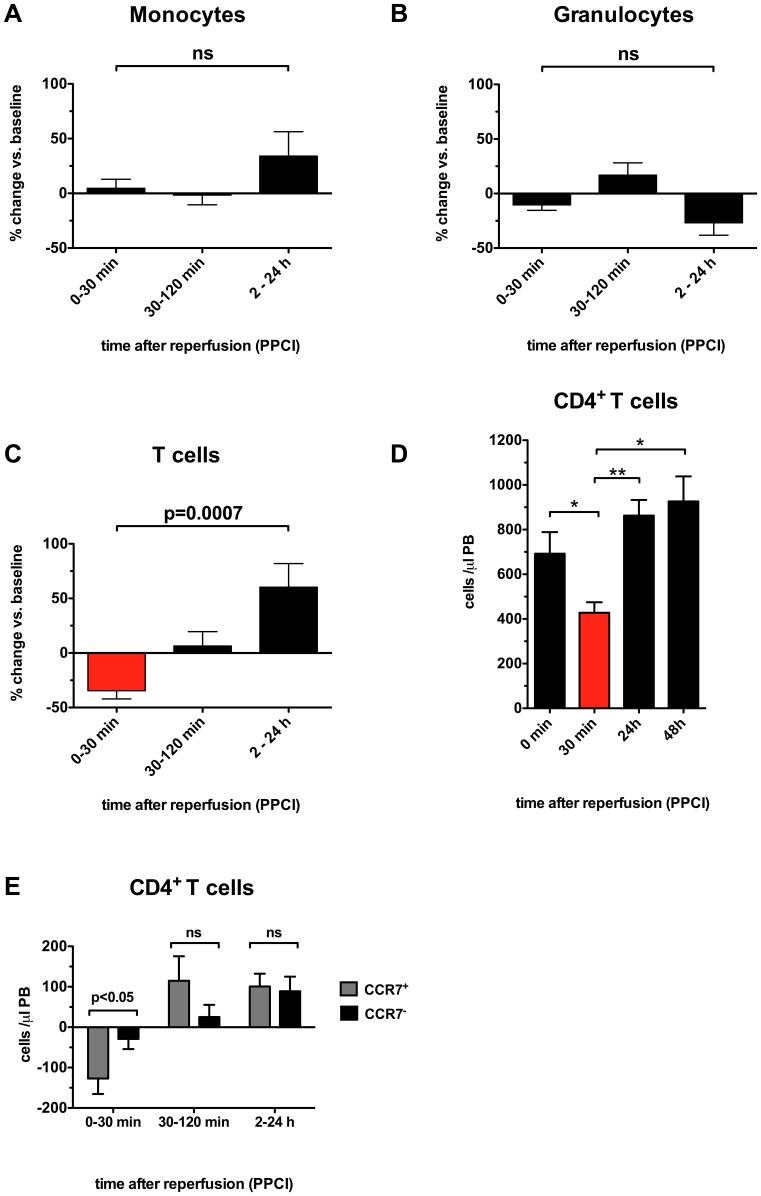
CD4^+^CCR7^+^ T cells are specifically depleted from peripheral blood during myocardial reperfusion. Time course pilot study showed no significant changes in numbers of monocytes (A) and granulocytes (B) following PPCI. In contrast, T cells decreased by 35% during the first 30 min following reperfusion showing significant relative increase within next 24 hours (C). The absolute cell count analysis revealed a significant decrease in CD4**^+^** T cells during the first 60 min following PPCI (D). This was mainly due to the specific depletion of circulating CD4**^+^**CCR7**^+^** cells within the first 30 min following reperfusion (E). *p<0.05, **p<0.01, ns not significant.

Given that 24 h after PPCI we saw signs of recent proliferation in the central memory T-cell compartment, the question about the cause remained. Potential explanations would include myocardial ischemia preceding PPCI, myocardial reperfusion, or simply general inflammation. We therefore looked at a second study population consisting of 17 patients undergoing PPCI. Blood samples were taken before reopening of the infarct-related artery, as well as after 30 min, 2 h and 24 h. From these data points we calculated the relative changes in the resulting time intervals. Surprisingly, monocytes (Figure 6A) and granulocytes (Figure 6B) did not show any significant changes following reopening of the infarct-related artery. In contrast, T cells decreased by 35% during the first 30 min following reperfusion, only to increase by 6% and 60% in subsequent time intervals (p = 0.0007, Figure 6C). Measurement of absolute numbers at each time point suggested that CD4**^+^** T cells might have been slightly reduced before PPCI (692±422 vs. 863±288 cells/µl, ns, Figure 6D), which in some cases might have been the result of spontaneous or pharmacological (aspirin, prasugrel) reperfusion before reaching the cathlab. This is reflected by several patients with TIMI1 flow prior to PPCI. However, the only significant drop in CD4**^+^** T cells occurred in the first 30 min following reperfusion (428±156 vs. 692±422 cells/µl, p<0.05, Figure 6D). Finally, we performed polychromatic flow cytometry on 9 of the 17 patients. After reviewing all clusters, we identified a subset of cells that was specifically depleted during early reperfusion. CD4**^+^**CCR7**^+^** T cells were reduced by 127±115 cells/µl in the first 30 min, while CD4**^+^**CCR7**^−^** T cells only decreased by 29±75 cells/µl in the same time interval (p<0.05, Figure 6E). CD4**^+^**CCR7**^+^** T cells, which also include central memory cells, already started increasing between 30 min and 2 h following reperfusion (115±183 cells/µl, Figure 6E). Altogether, this suggests an important role of CD4**^+^**CCR7**^+^** T cells during early reperfusion and potentially myocardial ischemia/reperfusion injury.

## Discussion

Primary PCI has led to a further decline in acute mortality in patients with acute myocardial infarction in recent years. Coronary anatomy as well as the time interval between the onset of ischemia to reopening of the infarct-related vessel represent two major predictors of infarct size and outcome, prompting ever more efficient networks to e.g. transport patients directly to 24 h PCI centers and reduce door-to-balloon-time. However, another major determinant of infarct size remains myocardial ischemia/reperfusion injury, affecting almost half of successfully treated patients [2]–[4]. While coronary angiography finds epicardial flow in the re-opened artery, cardiac MRI is frequently detecting signs of microvascular plugging and injury secondary to reperfusion, seen as microvascular obstruction (MVO) [19]. On a cellular basis there is no specific parameter at present, that allows for immediate assessment of reperfusion injury.

Myocardial I/R leads to a sustained inflammatory response, culminating in a dramatic influx of neutrophil granulocytes into the infarcting myocardium and their adherence to the endothelium. This in turn leads to capillary plugging, edema and a reduction in coronary flow. Studies in mouse models of I/R have identified lymphocytes as the cause of secondary influx of neutrophils. Specifically, CD4**^+^** T cells migrate to the infarcted myocardium following reopenening of the infarct vessel [8], [9]. Rag1 knockout mice, which lack T and B lymphocytes, are protected from myocardial I/R injury [9]. Infusion of CD4**^+^** T cells from interferon-γ knockout mice – but not wild type animals - into Rag1 ko mice fails to increase myocardial injury, suggesting that interferon-γ secreting CD4**^+^** T cells are major contributors to myocardial I/R injury in the mouse [9]. Interleukin-10, a cytokine that can suppress interferon-γ secreting CD4**^+^** T cells, has been shown to contribute to cardiac protection after MI in a mouse model [20]. It is conceivable though, that modelling myocardial I/R in the mouse does not exactly reflect the human situation, possibly due to 1) use of infarct models in otherwise young and healthy mice, 2) differences in the immune system, 3) a preponderance of lymphocytes as opposed to granulocytes among leukocytes (up to 70%) in most mouse strains, and 4) the presence of chronic ischemia pre infarction in some of the patients.

In humans, very little information exists regarding the potential role of leukocyte components during reperfusion. At least 5 studies have identified a high concentration of neutrophils and monocytes, low lymphocyte counts, and high neutrophil to lymphocyte ratio as predictors of major cardiovascular events or death following myocardial infarction [10], [21]–[23]. However, none of these studies has looked into early time intervals following PPCI or identified cell populations relevant to reperfusion with high accuracy. Polychromatic flow cytometry represents the only currently available tool for accurately detecting and characterizing of phenotypic leukocyte subset diversity in the human immune system. Although some recent reports presented a simultaneous flow cytometric quantification of up to 17 different fluorochromes and dyes [24]–[26], the reproducibility and practicability of such complex multiparameter measurements, especially in the context of large-scale clinical sample screening, often remain insufficient for widespread use. The limiting factors include lack of simple and reproducible cell processing and staining protocols, unavailability of appropriate instruments and/or on-site flow cytometry expertise (especially in smaller clinical centres), low time and cost efficiency of polychromatic flow cytometry measurements, as well as problems with a rapid and objective analysis of complex multiparameter data. In consequence, many of the potential advantages of multiparameter flow cytometry still remain poorly applicable for scientists and clinicians across different medical disciplines, including cardiovascular research.

Our study has provided 3 important results: We have shown for the first time that analysis of complex immunophenotypic patterns is possible even in patients with acute myocardial infarction, where two third of the patients are being treated out of hours. We managed to develop a standardized protocol for analysis of 13 parameters. Interassay variability was kept at 4%, allowing excellent comparability of spatial measurements. Flow-sorting of subpopulations from cryopreserved samples was simple as well, which allowed DNA isolation and telomere length measurements specifically in CD4**^+^** T cells. Second, we showed that a recently developed algorithm, which allowed hierarchical clustering analysis (HCA), enabled identification of differential cell populations. Finally, as an example for the practicability of our protocol, we identified a depletion of CCR7**^+^** CD4**^+^** T cells from peripheral blood during the first 30 min of reperfusion following PPCI. The chemokine receptor CCR7 has been identified as a key regulator of T-cell trafficking to secondary lymphoid organs [27]. However, CCR7 can also be involved in the abnormal recruitment of T cells from the peripheral blood to sites of acute inflammation [28]. CCR7 is expressed on naive, central memory and regulatory T cells [27]. CCL19 and CCL21 are the only ligands of CCR7 and both are expressed by endothelial cells [27]. Activation of the ligands leads to recruitment of CCR7-containing naive T cells to the endothelial layer in lymph nodes, but most likely in other locations as well. Interestingly, increased levels of CCL19 and CCL21 in atherosclerotic plaques have been found in stable and unstable angina [29]. Plasma levels of CCL21 increase up to 3-fold during the first 2 hours following PCI, and CCL19 levels peak at 24 h post PPCI [29]. Accordingly, genetic knockout of the CCR7 receptor in LDL receptor knockout mice attenuates atherosclerotic plaque development [27], [30]. Importantly, CCR7 signalling pathways in murine and human T cells are identical [27]. Given the temporal regulation of both CCR7 ligands during PCI as well as their role in T-cell trafficking to inflamed sites, it is conceivable that CCR7 and its two ligands could play an important role in T-cell migration to the infarcted heart during I/R following primary PCI.

Multiple clinical trials have attempted to target reperfusion injury. Antioxidants, reduction of intracellular calcium overload, antiinflammatory agents, adenosine, metabolic regulation, magnesium, nicorandil as well as therapeutic hypothermia, by large have failed to achieve significant reduction in infarct size [31] (for review, see [19]). So far, no trial has targeted T cells directly. However, the application of the immunosuppressive agent cyclosporin A prior to reperfusion has proven successful to reduce infarct size as measured by cardiac MRI and integrated troponin serum levels [32].

Our study identified for the first time significant T-cell shifts in patients with myocardial infarction following coronary reperfusion. The fact that these shifts did not occur in other leukocyte compartments, indicates a specific mechanism. However, it has to be pointed out that despite the „disappearence” of T-cells from the peripheral blood one cannot conclude that the remaining cells have entered the myocardium. While cell death is an unlikely explanation, redistribution of T-cells to the bone marrow or lymphatic tissues such as the spleen could certainly present a possibility. Future studies, including animal models and, e.g. measurements of the transcoronary gradients of the selected T-cell subsets in patients following reperfusion, may provide more mechanistic evidence. However, T-cell compartment shifts could be a surrogate marker for the extent of I/R-injury-related inflammatory response.

## Supporting Information

Materials S1 (PDF)
