# Supplementary material for: High-Throughput 13-Parameter Immunophenotyping Identifies Shifts in the Circulating T-Cell Compartment Following Reperfusion in Patients with Acute Myocardial Infarction
Source: PLoS One. 2012 Oct 16;7(10):e47155. doi: 10.1371/journal.pone.0047155 (PMC3473067; doi:10.1371/journal.pone.0047155)
Supplement: Materials S1 — (PDF) [file pone.0047155.s001.pdf]

Online supplementary methods

Study subjects

55 men with angiographically confirmed coronary heart disease were included into the study. Thirty-one patients with acute ST-elevation myocardial infarction (STEMI, mean age 56.1±5.7 years) and 24 stable patients with healed STEMI (at least 3 months after infarction, mean age 61±6 years) were analysed 24 hours following primary coronary intervention (PPCI) or routine control coronary angiography, respectively. None of the patients were affected by neoplastic, autoimmune or chronic infectious disease. All subjects with recent infections were also excluded. Eighteen healthy male volunteers were enrolled as controls (mean age 53.5±5.8 years). For the time-course substudy 17 patients with STEMI (mean age 59.7±9.5 years) were sequentially analysed within 48 hours following PPCI **(supplementary figure 1)**. The study protocol was approved by the institutional ethical committee of Newcastle University (REC 09/H0905/50), and written informed consent was obtained from all patients and healthy volunteers.

Blood Collection

80 ml or 20 ml (for the time-course substudy) of peripheral blood were carefully drawn by venipuncture into 10 ml EDTA collection tubes (BD Vacutainer, Cat. No. 366643, BD Biosciences, San Jose, CA, USA). Absolute leukocyte counts were determined and blood samples were maintained at 18-21°C (room temperature RT) until Ficoll gradient processing within 2 hours following collection.

Enumeration of peripheral blood leukocyte subsets

Absolute counts of peripheral blood granulocytes, monocytes and lymphocyte subpopulations were determined using BD TruCount flow cytometry assay (Cat. No. 340334, BD Biosciences). Briefly, 50µl of freshly drawn peripheral EDTA blood were added into TruCount tube using reverse pippeting and stained with 10 µl CD3/CD8/CD45/CD4 BD Multitest reagent (containing anti-CD3-FITC, anti-CD8-PE, anti-CD45-PerCP, and anti-CD4-APC monoclonal antibodies, Cat. No. 342417, BD Biosciences) and 5 µl of anti-CD14-Pacific Blue (Cat. No. MHCD1428, Invitrogen). Following 20 min incubation at room temperature in the dark, erythrocytes were lysed

for 15 minutes with 1500 µl ammonium chloride-based lysing buffer (BD PharmLyse, Cat. No. 555899, BD Biosciences). Data acquisition was performed on BD FACS Canto II flow cytometer (BD Biosciences) using FACSDiva software (BD Biosciences). Threshold was set on CD45 PerCp channel and 10000 of CD45 positive lymphocytes were counted for the stop gate. Spectral overlap between different channels was calculated automatically by the FACSDiva software after measuring single-colour compensation controls. Optimal compensation was achieved using antibody capture beads (Anti-Mouse Ig, κ CompBeads, Cat. No. 552843 BD Biosciences) and the corresponding conjugated antibodies (see below). Data was analyzed using FACS-Diva Software and absolute cell counts pro µl PB were calculated according to manufacturer's protocol.

PBMCs isolation, cell cryopreservation and storage ("PBMC Cryobank")

Peripheral blood mononuclear cells (PBMCs) were obtained after density gradient centrifugation using Ficoll-Hypaque (Biochrom, Cat. No. #L6115). PB blood was transferred from EDTA collection tubes into 50 ml conical tubes (BD Falcon) and gently resuspended with PBS in a ratio 1:1. Subsequently, 15 ml of Ficoll-Hypaque were placed at the bottom of a second 50 ml Falcon tube, and 35 ml of the blood/PBS-mix were then slowly layered above Ficoll. After being centrifuged for 20 min at 800xg at RT (without brake), the following layers were visible in the Falcon tube, from top to bottom: plasma with platelets and PBS, a yellow-whitish layer of mononuclear cells (PBMCs interphase), a clear layer of Ficoll-Paque, whitish layer of granulocytes and cell-debris covering the dark-red pellet of erythrocytes. The PBMC interphase was than carefully aspirated using a slow-speed automatic 10 ml pipette and transferred to a fresh Falcon tube. Following two washes with PBS/2mM EDTA PBMCs were counted with trypan blue using Neubauer haemocytometer, carefully resuspended in ice-chilled freezing medium containing RPMI 1640 (Cat. No. 21875034, Invitrogen) with 10% FBS (Cat. No. A15-151, PAA), 1% P/S (Penicillin/Streptomycin 5000Units/ml, Cat. No. 15070063, Invitrogen) and 10% DMSO (Cat. No. D2650, Sigma) (100µl medium pro $1x10^6$ cells) and aliquoted into 1 ml cryovials (Cat. No. CRY-960-070B, Thermo Fisher Scientific, Denmark). Cell aliquots were imediately frozen using 1ºC freezing containers with isopropyl alcohol ('Mr. Frosty' Cat. No. 5100-0001, Thermo Fisher Scientific), and stored at -80ºC

(Premium -86° Ultra-Low Temperature Laboratory Freezer, Cat. No. 539-107, New Brunswick Scientific, Edison, NJ, USA).

Antibodies and fluorochromes for 11-colour flow cytometry

Both primary and secondary antibodies were carefully titrated prior to use in flow cytometry experiments. Directly conjugated anti-CD3-QDot-605 and CD16-PacificBlue were purchased from Invitrogen; anti-CD4-AlexaFluor-700, CD8-APC-H7, CD27-APC, CD28-PerCpCy5.5 and CCR7-PE-Cy7 were from Becton Dickinson. Anti-KLRG1-AlexaFluor-488 antibody (clone 13F12) was generated as described previously. For indirect immunofluorescence purified mouse anti-human-CD57 (BD Biosciences) and biotin-labeled anti-PD1 (eBioscience) were used as primary antibodies. Secondary staining was performed with AlexaFluor-350-labeled Goat-anti-mouse (BD Bioscience) and Streptavidin-AlexaFluor532 (Invitrogen), respectively. For dead cell exclusion LIVE/DEAD® Fixable Aqua Dead cell dye (Invitrogen) has been included into PBMC staining panel **(see supplementary figure 2 and supplementary table 2)**.

Cell staining for 11-colour flow cytometry

Frozen PBMC aliquots ($2x10^6$ PBMC/aliquot) were quickly thawed in water bath (37ºC for 1 min) and immediately transferred into polystyrene round bottom 12 x 75 mm Falcon tubes (BD Biosciences). After adding 1 ml wash buffer containing PBS (Cat. No. 18912-014, Gibco), 2mM EDTA and 0,1% azide, samples were washed once using automatic cell washer (BD Lyse Wash Asistant, Cat. No. 337146). This high-throughput washing technique provides much better cell recovery while maintaining higher viability of thawed PBMCs as compared with traditional centrifuge-based washing. Following primary wash step, the cell viability and cell numbers were analysed using video imaging system with automated trypan blue exclusion protocol (Vi-Cell XR Cell Viability Analyzer, Beckman Coulter,). $1x10^6$ PBMCs were then transferred into new FACS tube and the total sample volume ('stain volume') was adjusted up to 100 µl with PBS/2mM EDTA. For staining, master mix containing primary antibodies was added into tubes and samples were incubated light protected for 20 minutes at RT. Quantum-dot-conjugated antibodies (CD3-QDot-605) were added separately into samples, prior to addition of the primary antibody mix. This ensured optimal staining results as the used nanocrystals turned to get unstable

when premixed with other primary antibodies **(supplementary figure 3)**. Following incubation with primary antibodies, 1 ml of wash buffer per sample was added and samples were washed three times using lyse wash assistant. Following wash steps, appropriate amounts of secondary antibodies (2 µl of Streptavidin-AF532 and 5 µl of rat anti-mouse-AF350) and 1 µl of Aqua Dye were added into the total sample volume of 300 µl. Samples were then incubated light protected for another 20 minutes at RT. Following incubation, 1 ml of wash buffer per sample was added and cells were washed again three times using lyse wash assistant. Following these final wash steps, unfixed samples (total end volume - 300 µl) were immediately subjected to analysis by flow cytometry.

Flow cytometry data acquisition

Samples were measured in our flow core facility on a BD LSR II cytometer (see below for detailed description) using BD FACSDiva acquisition software. PMT voltages and compensation values were set as described below. At least 100000 viable cell events per sample were acquired.

Flow cytometry data analysis

The analysis of acquired data was performed using MATLAB (for Hierarchical Cluster Analysis, see below) or FlowJo software (Version 9.4.1 for Macintosh). Two-dimensional plots (pseudo-color or dot plot) were created using biexponential transformation and sequential gating of viable T cells was performed according to the model of T-cell memory differentiation as described by Sallusto and Romero [2,3,4] **(see supplementary figure 4)**.

Instruments setup

The Becton Dickinson LSR II flow cytometer was equipped with 5 spatially and time delayed lasers: blue (488nm), violet (407nm), UV (355nm), red (638nm) and green (532nm). The detailed configuration of our LSR II can be seen below **(see supplementary table 1)**. Prior to any analysis the instrument was checked for correct laser delay settings and fluidics stability by consistency of results using cytometer tracking and set up beads using an acceptable tolerance of +/- 10 volts (Becton Dickinson), and single peak Spherotech Ultra Rainbow beads.

PMT voltage settings

At the time of experimental set up the PMT voltages were adjusted for each fluorochrome with the objective of optimizing the signal to noise ratio, based on single stained samples.

Spectral compensation

Spectral overlap between different fluorochromes was calculated automatically by the FACSDiva software. For this step we used the voltages determined by measuring single-colour fluorophores, as described above under PMT voltage settings.

There were 2 steps to the compensation set up

1. The specific test antibodies were bound to antibody capture beads (Becton Dickinson. For the unconjugated antibodies those tubes were washed then the secondary fluorochrome reagent added, after a second 20 minute incubation all tubes were washed.
2. For the Aqua dead cell dye a dead cell compensation control tube (aged sample) consisting of cells was acquired and the compensation for this was done seperately from the automatic compensation matrix , but then added to the final compensation values for the experiment.

Verification of the compensation values was achieved by appending all of the individual compensation beads into one list mode file so as to ensure the correct mean fluorescence values have been achieved with respect to each fluorochrome. Compensation matrix used in the study can be seen below **(supplementary table 3)**. Only minor experiment-to-experiment adjustments of this set up were needed in the course of the present study.

Staining/gating controls

For optimal two-dimensional gating setup 'fluorescence minus one' (FMO) controls were included in each experiment. FMO controls contained all fluorochromes but the one of interest. This strategy enabled most accurate gating of 'postive' and 'negative' events, especially for weakly expressed antigens that often give 'continuous populations', such as PD-1 or CCR7. For example, FMO control sample for CCR7 was stained with Aqua dye and all antibody-conjugates except anti-CCR7-PE-Cy7 **(see supplementary figure 5)**.

Dead cell exlusion in 11-colour flow cytometry panel

Staining of cryopreserved PBMCs required incorporation of an additional dye for dead cell exclusion. Among tested cell viability reagents, Aqua dye provided best resolution between dead and viable cells with only minimal requirement for spectral compensation and without any putative effects on other reagents included in the sample **(supplementary figure 6A)**. **Supplementary figure 6B** illustrates exemplarily a decrease in sensitivity for detection of the tandem conjugate CD28-PerCp-Cy5.5 upon addition of another dead cell marker, propidium iodide (PI) into sample stained with the full antibody panel. **Supplementary figure 7** demonstrates the importance of additional staining with viability dye for a proper dead cell exclusion. Gated dead cells appeared positive for all conjugates used in the panel and could not be properly distinguished from viable lymphocytes by their light scatter properties (e.g. SSC vs. FSC).

Variability of the multiparameter flow-cytometry panel

The variability of the multiparameter immunophenotyping method was calculated by analysing the repeated measurements of the frozen PBMC aliquots from the same healthy donor. We used 28 independent measurements, each performed on a different day. The values for Inter-experiment variability for the major T-cell subsets are shown in the **supplemetary table 4**.

Hierarchical Cluster Analysis (HCA) of flow cytometry data

Following data acquisition, files were exported from DIVA and saved as .fcs version 3.0 files. The analysed parameters were eleven colour channels (KLRG1, CD3, CD28, CCR7, CD45RA, CD57, CD27, CD4, CD8, PD1, Aqua Dye) and 3-4 parameters based on forward and side scatters (FS-A, SS-A, SS-H, (SS-W)). The raw or CD3 positive cells pre-gated data were extracted from .fcs files and imported into MATLAB environment (MATLAB, MathWorks, Natick, MA), where all subsequent steps were carried out. Compensation matrix (as present in .fcs files) was applied to the data followed by biexponential-like transformation and normalisation (zscore). After this the data were ready for HCA, which was performed using our new algorithm described previously [5] .

HCA serves as an alternative to gating, providing hierarchy of cells present in flow cytometry sample. The hierarchy is present in a form of dendrogram (hierarchical

tree) and heatmap (dendroheatmap, **supplementary figure 8**). Heatmap is a table of flow data with cells in rows and measured parameters in columns of the table. Cells are ordered based on HCA (compared to originally random order) and levels of measured parameters are colour coded, i.e. red-positive cells, blue-negative cells. Brunches of dendrogram are "cutted" (manually or automatically) into clusters, which are equivalents to gated cell populations. Dendroheatmap allows for selection of clusters on different levels in different parts of dendrogram. For example apart from main cellular populations selected on one level **(supplementary figure 8)** also cellular debris or doublets could be "cutted-out" on higher level in the same sample **(supplementary figure 9)**.

8-colour flow sorting of CD4 T Cells (Aria II)

$3x10^7$ cryopreserved PBMCs were used for fluorescence-activated cell sorting. Cell aliquots were thawed and processed as described above. Prior to staining, cells were evaluated for size, viability and concentration using the Vi-Cell analyzer. PBMC staining was performed using carefully pre-titrated antibody mix containing anti-human-CD3-FITC, CD4-PE-Cy7, CD8-APC-H7, CCR7-PE CD45 (BD Bioscience), CD45-Pacific Blue and Aqua dead cell dye (Invitrogen). Cell sorting was performed in our flow core facility on a BD FACS AriaII cell sorter equiped with 4 lasers: 638nm Red, 355nm UV, 405nm Violet and 488nm saphire lasers. The sorting conditions were 70micron nozzle at 70 psi, using a high purity mask and acquisition speed below 10000 events per second. $3\text{-}5x10^5$ $CD4^+$ T cells were sorted directly into 1.5 ml eppendorf tubes. The sorted aliquots were spun down and the flow buffer supernatant was completely removed with a vacuum suction. Dry T-cell pellets were stored at -80ºC freezer until further processed with DNA isolation protocol (see below).

DNA isolation and telomere length RT-PCR assay

DNA was extracted from sorted $CD4^+$ T cells with the QIAamp DNA Mini Kit (Cat. No. 51304, Qiagen Ltd, Crawley, UK). DNA concentration and quality were monitored by agarose gel electrophoresis. Samples were discarded if DNA degradation (smear <20 kb) was visible. Telomere length was measured as the ratio of the starting quantity for telomeres versus the starting quantity for the single copy gene of glyceraldehyde-3-phosphate dehydrogenase (as control) by quantitative real-time

polymerase chain reaction (PCR) [6] with modifications as described previously [7]. Measurements were performed in quadruplicates. Three DNA samples with known telomere lengths (3.0, 5.5 and 9.5 kb pairs) were run as internal standards together with each batch of 16 study samples to convert the ratios of starting quality into telomere lengths in base pairs. The intra-assay coefficient of variation for this PCR method in our lab is 2.65% and the inter-assay coefficient of variation is 5.12%.

Statistical analysis

In the text, data are reported as mean±SE. Comparison of 3 means was performed by ANOVA, followed by Tukeys' post-hoc test. Comparison of 2 groups was calculated using an unpaired t-test, if normal probability plots (P-P plots) demonstrated approximate normality. All statistical tests were performed using GraphPad Prism version 5 for Macintosh (www.graphpad.com).

[1] Marcolino I, Przybylski GK, Koschella M, Schmidt CA, Voehringer D., Schlesier M, and Pricher H. Frequent expression of the natural killer cell receptor KLRG1 in human cord blood cells: correlation with replicative history. Eur J Immunol 2004, 34:2672.

[2] Sallusto F, Lenig D, Forster R, Lipp M, Lanzavecchia A. Two subsets of memory T lymphocytes with distinct homing potentials and effector functions. Nature 1999, 401: 708-712.

[3] Romero P, Zippelius A, Derhovanessian E, et al. Four functionally distinct populations of human effector-memory CD8+ T lymphocytes. J Immunol 2007, 178: 4112-4119.

[4] Koch S, Larbi A, Petrie J et al. Multiparameter flow cytometric analysis of CD4 and CD8 T cell subsets in young and old people. Immun Ageing 2008, 5:6.

[5] Fišer K, Sieger T, Schumich A et al. Detection and monitoring of normal and leukemic cell populations with hierarchical clustering of flow cytometry data. Cytometry A 2012, 81: 25-34.

[6] Cawthon RM, Smith KR, O'Brien E et al. Association between telomere length in blood and mortality in people aged 60 years or older. Lancet 2003; 361: 393–5.

[7] Martin-Ruiz C, Saretzki G, Petrie J et al. Stochastic variation in telomere shortening rate causes heterogeneity of human fibroblast replicative life span. J Biol Chem 2004; 279: 17826–33.

Supplementary figures

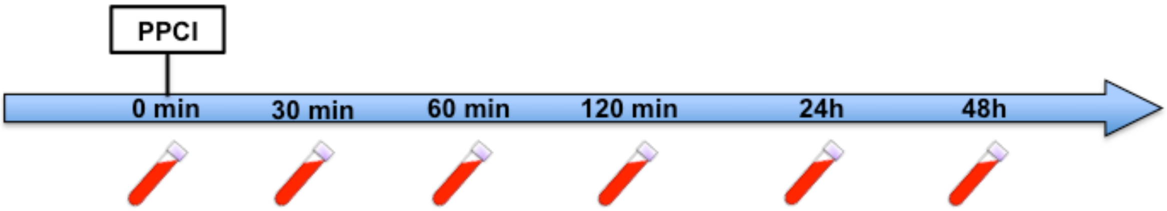

Supplementary figure 1.

Flow chart representing set up of the time course substudy.

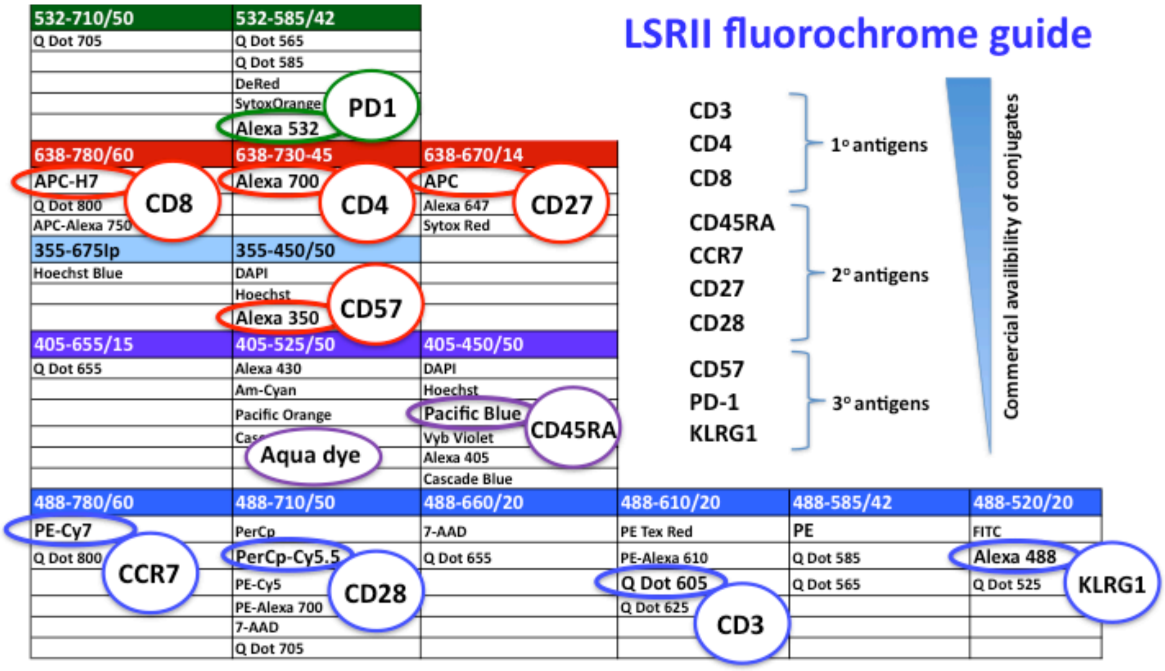

Supplementary figure 2.

LSR II flow cytometer fluorochrome guide used for construction of the 11-colour panel.

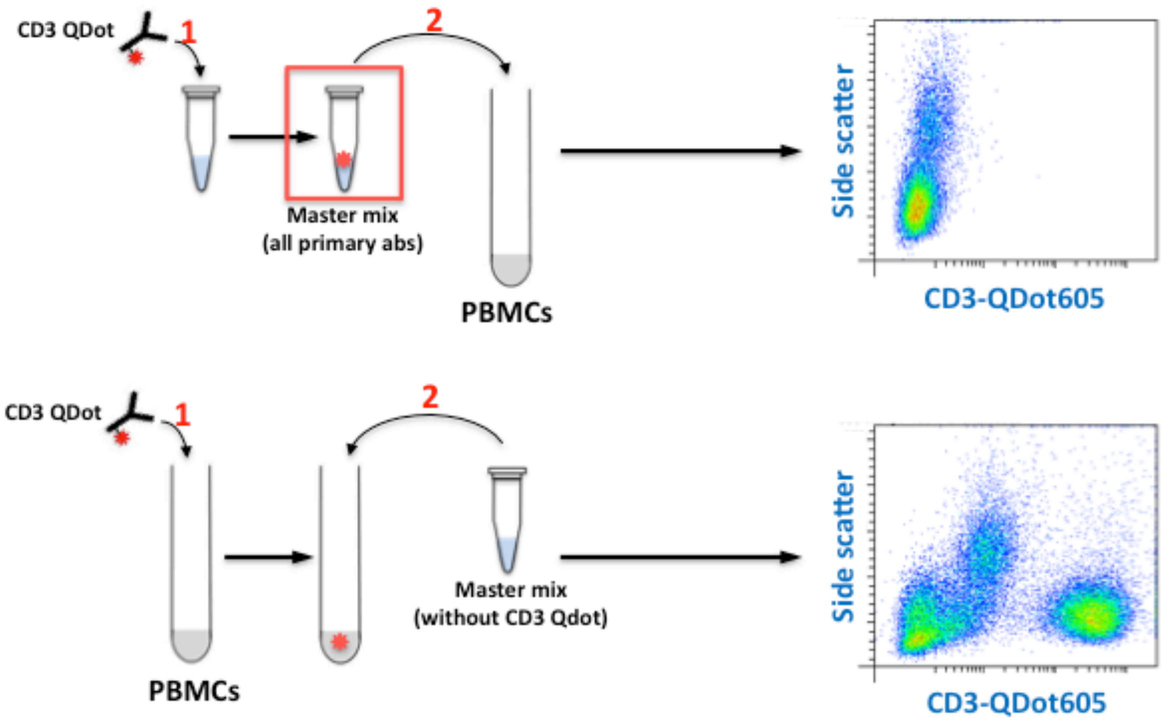

Supplementary figure 3.

Troubleshooting regarding incorporation of quantum dot nanocrystals into the 11-colour flow cytometry panel (own observations). When QDots were added in a single stained sample it gave a good signal to noise, when premixed with the other antibodies (master mix), prior to cell staing, the signal disappeared (upper panel). This could be finally resolved by 1) doubling the concentration of QDots (following titration set up, data not shown) and 2) adding QDots separately into cell sample prior to adding the remaining primary antibodies (master mix without CD3 QDot, lower panel).

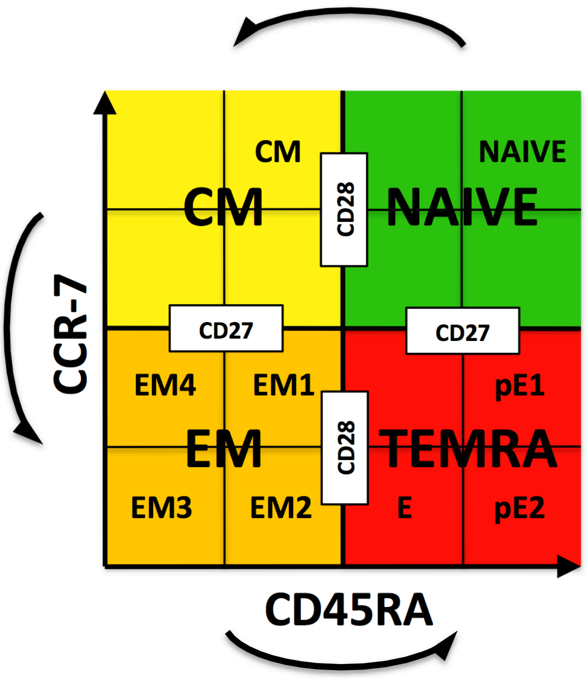

Supplementary figure 4.

Model of T-cell differentiation as described by Sallusto et al. and Romero et al.

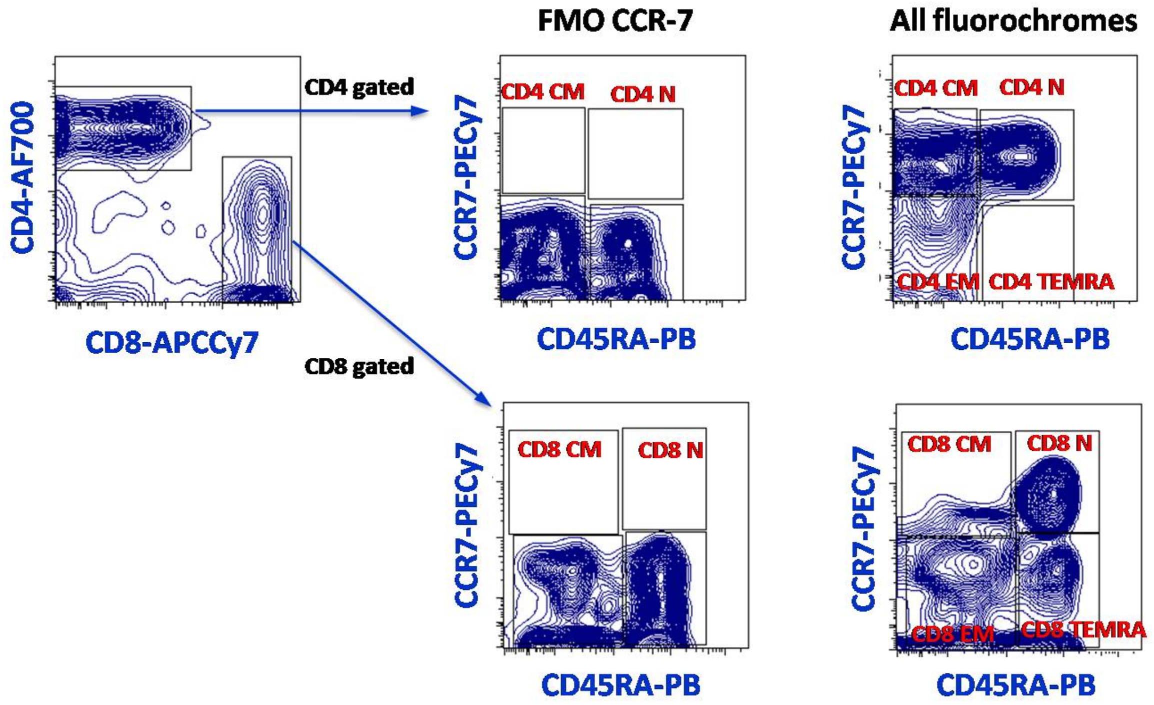

Supplementary figure 5.

Example of fluorescene-minus-one (FMO) controls to determine the positive/negative boundary for CCR7 expression on CD4^{+} and CD8^{+} T-cell subpopulations.

A

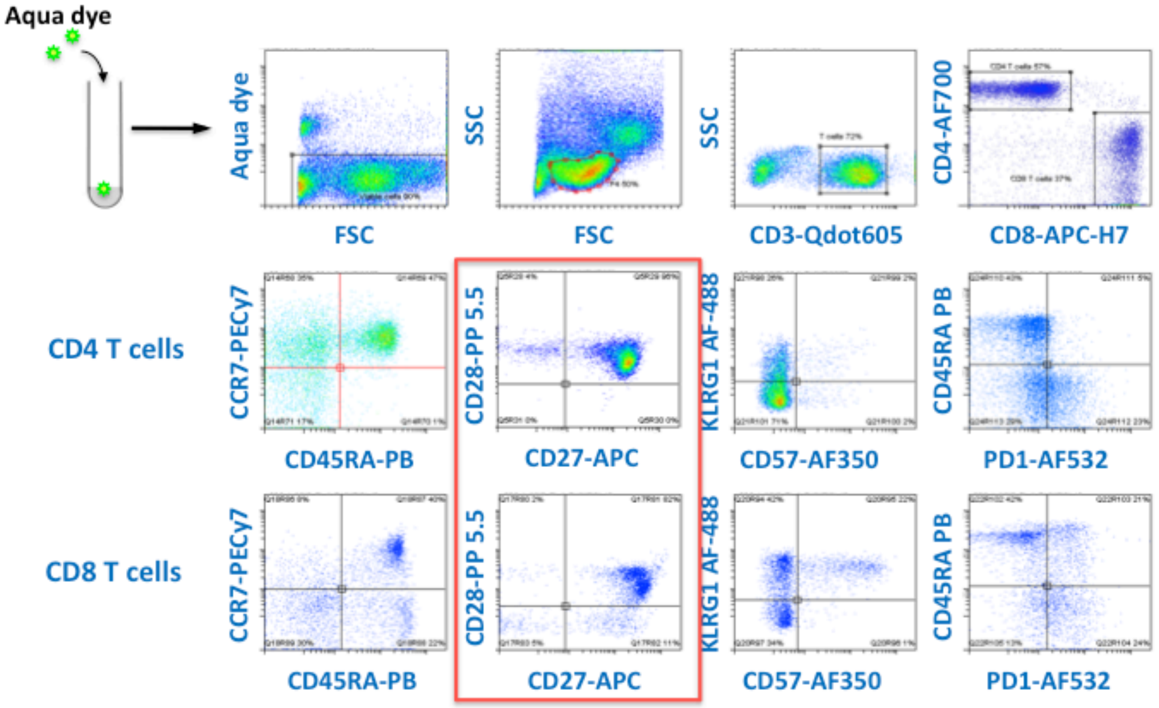

B

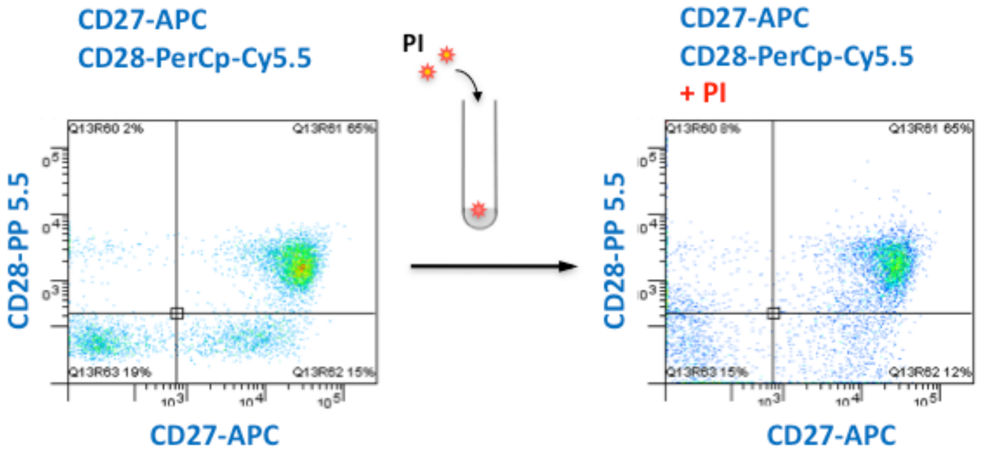

Supplementary figure 6.

Implementation of the dead cell staining into a 11-colour flow panel. The upper panel shows thawed PBMCs stained with 10 monoclonal antibodies and Invitrogen Live/Dead Aqua dye **(A)**. The lower panel shows deterioration PerCpCy5.5 (CD28) fluorescence following addition of propidium iodide into the sample (gate on CD8+ T cells) **(B)**.

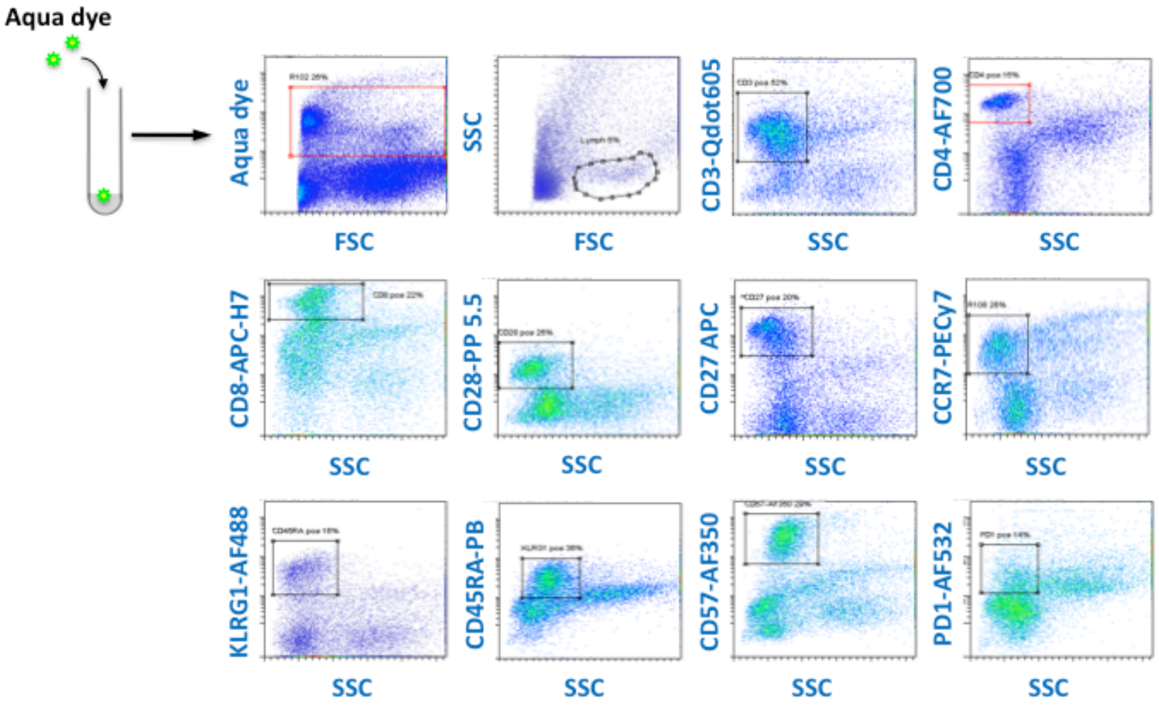

Supplementary figure 7.

Nonspecific antibody binding by dead cells. The single dot plot panels show the gated Aqua dye postive cells.

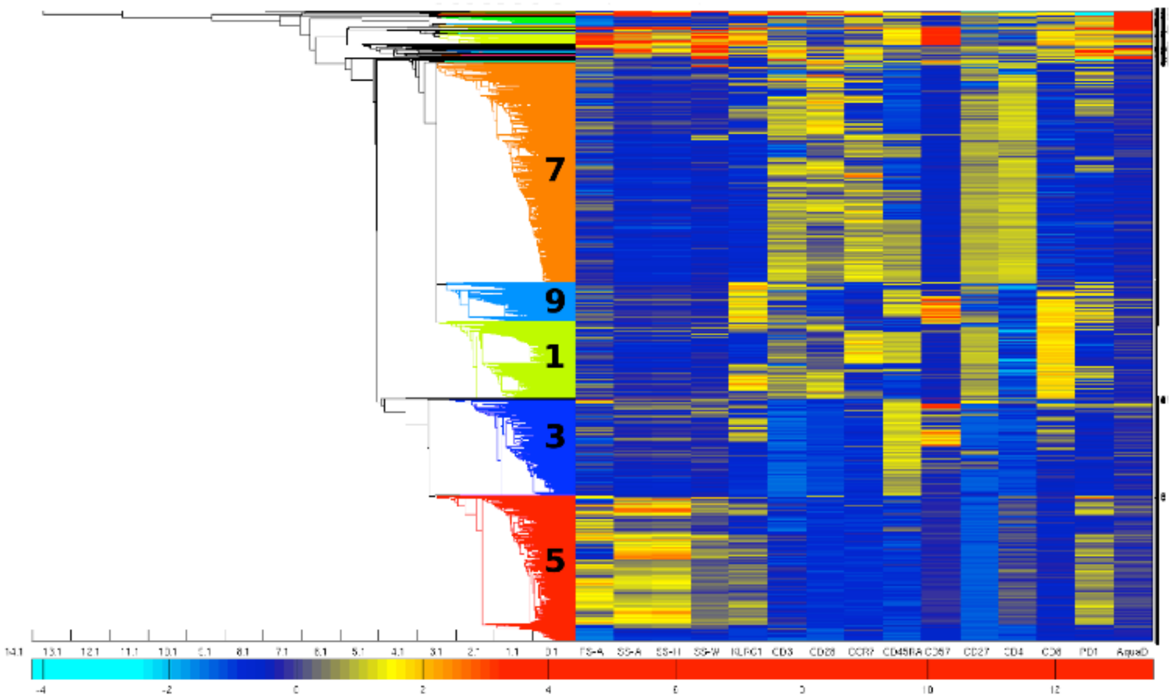

Supplementary figure 8.

Dendroheatmap. HCA of healthy donor sample (10000 ungated events, 15 parameters). Heatmap has cells as rows and measured parameters as columns with level of parameter color-coded: red-high, yellow-medium, blue-low, cyan-negative. Five major clusters were selected: 7, 9, 1, 3 and 5.

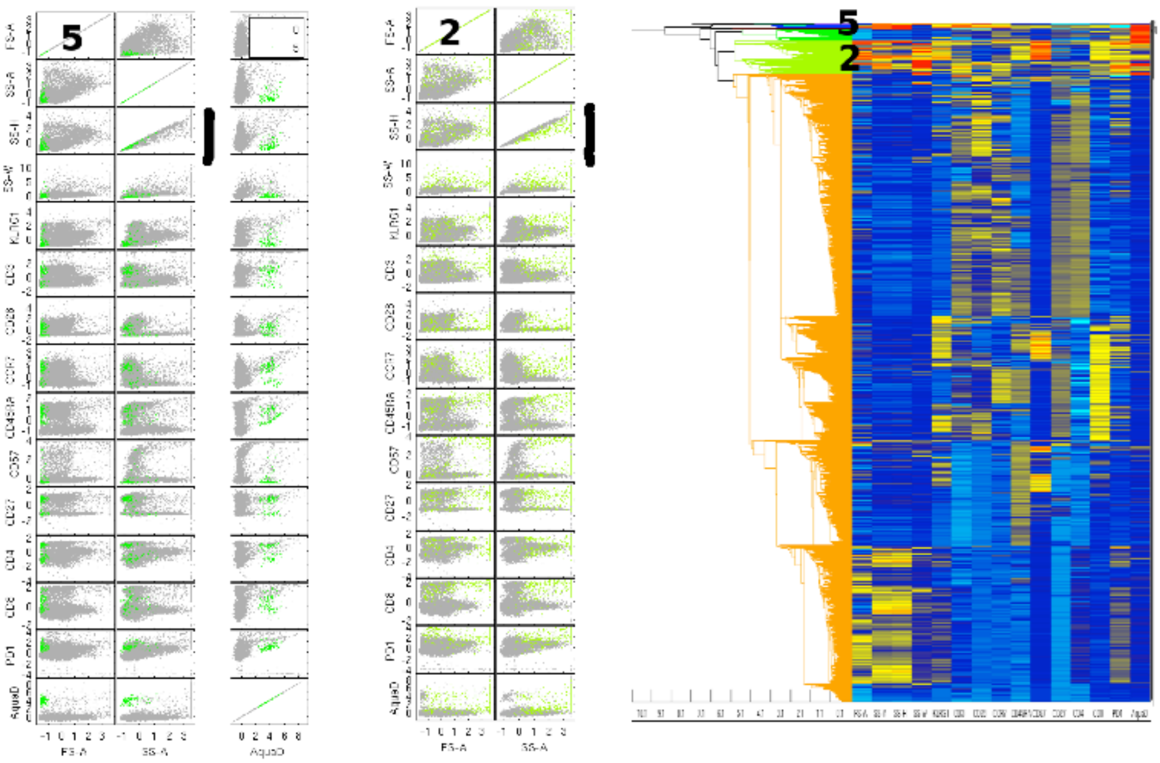

Supplementary figure 9.

DendroHeatmap and selection of scatterplots of two clusters on different dendrogram cutting level. Example of higher cutting level to show separation of Aqua Dye+ dead cells (cluster 5) and doublets (cluster 2). Remaining orange cells could be then separated into clusters in supplementary figure 8 (dendroheatmap).

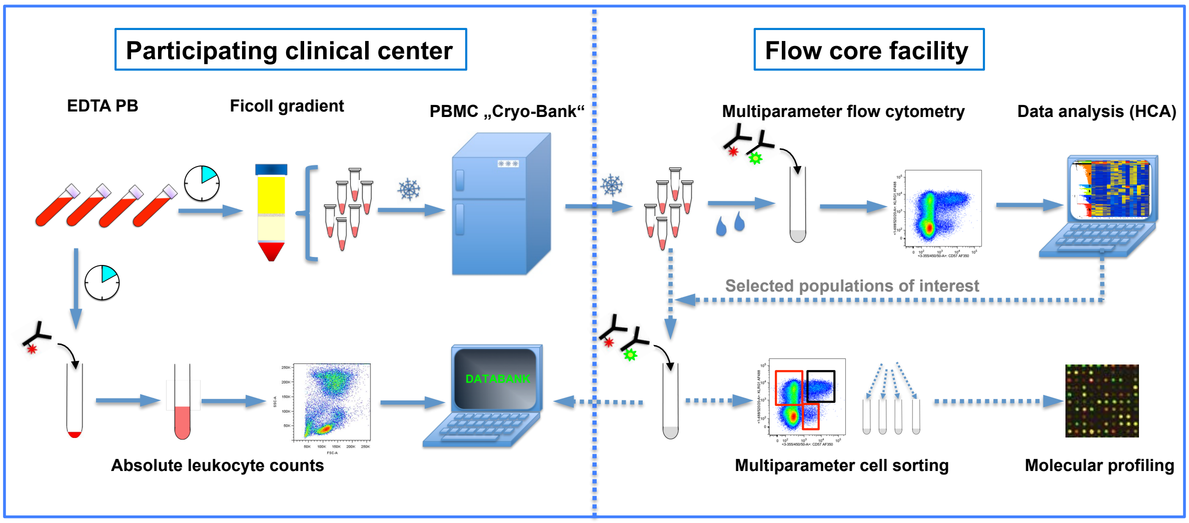

Supplementary figure 10.

High-Throughput multiparameter immunophenotyping and hierarchical cluster analysis as a novel biomarker screening tool for multicenter trials in cardiology. The selected PBMC subsets of interest may be additionally purified from remaining frozen aliquots using meultiparameter flow sorting and subjected to further molecular profiling (e.g. telomere length analysis, gene- or micro-RNA arrays, etc.).

Supplementary tables

Green	685	710/50
532 nm	Blank	585/42
Red	750	780/60
638nm	690	730/45
	Blank	670/14
UV		
355nm		
	635lp	675lp
	Blank	450/50
Violet	630	655/15
405nm	505	525/50
	Blank	450/50
Blue	735	780/60
488nm	685	710/50
	650	660/20
	600	610/20
	550	575/26
	505	520/20
	Blank	488/10

Supplementary table 1.

The detailed configuration of the used LSR II. The order of laser cell interrogation is Blue, Violet, UV, Red then Green.

Specifity	Fluorochrome	MoAb Clone	MoAb isotype	Manufacturer	Catalog number	Laser (nm)	Bandpass filters (nm)	Long-pass dichroic mirror (nm)
**CD3**	**QDot-605**	UCHT-1	Mouse IgG1	Invitrogen	Q10054	Blue(488)	610/20	600
**CD4**	**Alexa Fluor 700**	RPA-T4	Mouse IgG1, κ	BD	557922	Red(638)	730/45	690
**CD8**	**APC-H7**	SK1	Mouse IgG1, κ	BD	560273	Red(638)	780/60	750
**CD45RA**	**Pacific Blue**	MEM-56	Mouse IgG2b	Invitrogen	MHCCD45RA28	Violet(405)	450/50	blank
**CCR-7**	**PE-Cy7**	3D12	Rat IgG2a, κ	BD	557648	Blue(488)	780/60	735
**CD27**	**APC**	L128	Mouse IgG1, κ	BD	337169	Red(638)	670/14	blank
**CD28**	**PerCp-Cy5.5**	L293	Mouse IgG1, κ	BD	337181	Blue(488)	710/50	685
**KLRG1**	**Alexa Fluor 488**			-	-	Blue(488)	530/30	blank
**CD57**	**(Purified)**	TBO1	Mouse IgM	eBioscience	16-0577	-	-	-
**-**	**Alexa Fluor 350**		Goat anti-mouse IgG (H+L)	Invitrogen	A11045	UV(355)	450/50	blank
**PD-1 (CD279)**	**(Biotin)**	J105	Mouse IgG1, κ	eBioscience	13-2799	-	-	-
**-**	**Alexa Fluor 532**	-	(Streptavidine)	Invitrogen	S-11224	Green(532)	585/42	blank
**Dead cells**	**Aqua dye**	-	-	Invitrogen	L34957	Violet(405)	525/50	505

Supplementary table 2.

Monoclonal antibodies and fluorochromes used in the 11-colour staining panel.

	Alexa 488	Q Dot 605	PerCp-Cy5.5	PE-Cy7	PacifiC Blue	Alexa 350	APC	Alexa 700	APC-H7	Alexa 532	Aqua Dye
Alexa 488	100.00	17745,00	1.37	0.44	0.04	0.00	0.00	0.00	0.03	2.33	0.32
Q Dot 605	0.00	100.00	0.20	0.05	0.00	0.00	0.04	0.00	0.00	108.91	0.00
PerCp-Cy5.5	0.02	0.02	100.00	50.08	0.00	0.00	27.64	71.07	29.70	0.00	0.00
PE-Cy7	0.07	0.28	0.69	100.00	0.00	0.00	0.08	0.62	8.45	6.42	0.00
PacifiC Blue	0.02	0.00	0.00	0.00	100.00	1.68	0.01	0.00	0.00	0.00	6.61
Alexa 350	0.03	0.00	0.00	0.026	21.46	100.00	0.00	0.00	0.00	0.00	1.10
APC	0.00	0.00	0.58	0.26	0.00	0.00	100.00	57.77	18.18	0.00	0.00
Alexa 700	0.01	0.01	1.40	0.80	0.00	0.00	0.97	100.00	29.37	0.00	0.00
APC-H7	0.00	0.00	0.01	1.67	0.01	0.00	0.87	4.09	100.00	0.00	0.00
Alexa 532	0.07	2.96	0.71	0.23	0.00	0.00	0.00	0.00	0.00	100.00	0.00
Aqua Dye	3.00	2.60	1.80	1.00	18.90	6.20	3.10	2.10	0.00	5.90	100.00

Supplementary table 3.

Spectral compensation matrix used in the study.

Experiment date	Viable cells %ALL	Lymph %Viable Cells	T cells % Lymph	CD4+ % T cells	CD4 NAIVE %CD4	CD4 CM %CD4	CD4 EM %CD4	CD4 TEMRA %CD4
02. Aug 10	94.1	92.5	67.7	58.8	51.1	31.2	16.1	1.6
03. Aug 10	97.2	87.1	67.9	57.7	44.9	37.6	16.7	0.8
04. Aug 10	96.4	79.9	73.1	64.5	53.7	28.5	16.0	1.8
04. Aug 10	84.1	75.3	74.7	67.6	49.6	31.4	17.6	1.4
09. Aug 10	95.1	88.5	73.3	64.6	54.5	27.8	15.8	1.9
09. Sep 10	95.3	91.7	67.2	60.3	53.7	25.0	18.8	2.5
16. Sep 10	96.8	78.8	77.4	63.2	56.2	27.8	14.6	1.4
17. Jun 10	96.7	71.1	72.2	57.7	52.3	31.0	15.5	1.3
21. Jul 10	96.6	68.4	78.3	61.8	49.2	28.9	19.8	2.1
23. Aug 10	96.5	86.6	72.1	65.4	55.4	27.5	15.3	1.8
24. Jun 10	96.6	77.5	65.6	59.8	47.5	34.0	17.4	1.1
25. Aug 10	96.4	79.0	75.3	60.8	53.0	28.6	16.9	1.4
03. Aug 10	97.2	87.5	67.1	58.0	46.0	34.2	18.5	1.3
04. Aug 10	96.5	83.3	72.0	64.4	53.8	28.9	15.6	1.7
10. Aug 10	86.0	86.8	63.8	53.7	52.4	30.7	15.6	1.3
12. Aug 10	97.5	84.7	74.6	64.7	54.1	28.1	16.3	1.5
24. Aug 10	96.8	82.1	78.4	63.9	52.9	30.3	15.4	1.3
26. Aug 10	97.8	80.5	75.1	62.8	52.1	32.2	14.5	1.1
31. Aug 10	97.1	84.1	73.7	61.3	51.6	28.7	18.1	1.6
03. Sep 10	95.3	84.4	68.1	54.7	50.3	30.5	17.9	1.4
07. Sep 10	94.8	87.3	67.7	57.4	53.8	30.0	15.0	1.3
08. Sep 10	95.2	84.4	68.3	58.2	47.6	35.0	16.3	1.0
10. Sep 10	92.6	76.5	48.8	61.2	54.7	28.2	15.9	1.2
13. Sep 10	95.4	83.4	68.1	56.8	49.6	30.2	18.6	1.6
14. Sep 10	96.8	80.1	73.3	64.1	55.0	29.9	13.9	1.3
15. Sep 10	94.1	85.1	72.4	65.0	55.7	28.4	14.0	1.9
23. Sep 10	96.0	83.5	66.8	57.7	49.7	30.6	18.3	1.3
27. Sep 10	93.4	88.3	64.0	59.8	53.5	29.5	15.8	1.2
MEAN:	95.2	82.8	70.3	60.9	52.0	30.2	16.4	1.5
STD DEV:	3.1	5.7	5.9	3.5	3,0	2.6	1.5	0.4
CV:	3.3%	6.8%	8.4%	5.8%	5.8%	8.7%	9.4%	23.8%

Supplementary table 4.

Intra-panel variability for frequencies of the major $CD4^+$ and $CD8^+$ T-cell subsets.
